# Executive Functions of Swedish Counterterror Intervention Unit Applicants and Police Officer Trainees Evaluated With Design Fluency Test

**DOI:** 10.3389/fpsyg.2021.580463

**Published:** 2021-05-11

**Authors:** Torbjörn Vestberg, Peter G. Tedeholm, Martin Ingvar, Agneta C. Larsson, Predrag Petrovic

**Affiliations:** ^1^Department of Clinical Neuroscience, Karolinska Institutet, Stockholm, Sweden; ^2^Department of Physiology and Pharmacology, Karolinska Institutet, Stockholm, Sweden; ^3^Center for Cognitive and Computational Neuropsychiatry, Karolinska Institutet, Stockholm, Sweden

**Keywords:** executive functions, police, counterterror intervention unit applicants, cognitive flexibility, Delis–Kaplan Executive Function System, Design Fluency Test, special forces

## Abstract

Executive functions (EF) represent higher order top-down mechanisms regulating information processing. While suboptimal EF have been studied in various patient groups, their impact on successful behavior is still not well described. Previously, it has been suggested that design fluency (DF)—a test including several simultaneous EF components mainly related to fluency, cognitive flexibility, and creativity—predicts successful behavior in a quickly changing environment where fast and dynamic adaptions are required, such as ball sports. We hypothesized that similar behaviors are of importance in the selection process of elite police force applicants. To test this hypothesis, we compared elite police force applicants (*n* = 45) with a control group of police officer trainees (*n* = 30). Although both groups were better than the norm, the elite police force applicants had a significantly better performance in DF total correct when adjusting for sex and age [*F*(1,71) = 18.98, *p* < 0.001]. To understand how this capacity was altered by stress and tiredness, we re-tested the elite police force applicants several days during an extreme field assessment lasting 10 days. The results suggested that there was a lower than expected improvement in DF total correct and a decline in the DF3-subtest that includes a larger component of cognitive flexibility than the other subtests (DF1 and DF2). Although there was a positive correlation between the baseline session and the re-test in DF3 [*r*(40) = 0.49, *p* = 0.001], the applicants having the highest scores in the baseline test also displayed the largest percentage decline in the re-test [*r*(40) = −0.46, *p* = 0.003]. In conclusion, our result suggests that higher order EF (HEF) that include cognitive flexibility and creativity are of importance in the application for becoming an elite police officer but relatively compromised in a stressful situation. Moreover, as the decline is different between the individuals, the results suggest that applicants should be tested during baseline conditions and during stressful conditions to describe their cognitive capacity fully.

## Introduction

*Executive functions (EF)* is an umbrella term to describe the cognitive processes that regulate thought and action, and allow the individual to dynamically adapt to a changing environment (Friedman et al., 2006). EF involve planning, selective and sustained attention, inhibition, multi-tasking, cognitive flexibility, ability to deal with novelty, and problem-solving (Chan et al., 2008). Large-scale brain networks are involved in EF with prefrontal structures especially in focus (Kerns et al., 2004; Tanji and Hoshi, 2008; Mansouri et al., 2009; Stuss, 2011; Cieslik et al., 2015; Koechlin, 2016).

It has been suggested that EF can be sub-divided into *core EF* (CEF) and *higher order EF* (HEF) (Luciana et al., 2005; Diamond, 2013). While CEF include simple working memory, cognitive flexibility, and inhibitory control (Diamond, 2013), HEF are associated with planning, reasoning, and problem-solving (Diamond, 2013). The difference between CEF and HEF is related to the demands of the task, and HEF typically include several CEF simultaneously used for fast and accurate planning and problem-solving (Luciana et al., 2005; Diamond, 2013; Krenn et al., 2018). For example, holding information online to make a simple decision is more related to CEF. Conversely, maintaining and manipulating information in order to strategically organize goal-oriented behavior is more related to HEF. The level of CEF develops to its full capacity earlier in the lifespan than HEF, mostly before early adolescence (Luciana et al., 2005; Best and Miller, 2010).

Previously, research has focused on the relation between deficits of EF and behavior (Luria, 1980), development of EF (Anderson et al.,2001a,b), and their biological correlates in the brain (Stuss, 2011). Deficits in EF have been studied in disorders like ADHD, autism, and traumatic brain injury (Goldstein and Naglieri, 2014). EF are normally distributed among a general population, and disorders like ADHD may be thought of as low EF capacity in this distribution (Das et al., 2012; Crosbie et al., 2013; Petrovic and Castellanos, 2016; Bayard et al., 2018). The impact of the EFs on the other side of the continuum is still not fully explored.

A high EF capacity may be of importance in many different professions. We have, for example, previously postulated that well-developed EF are important in elite team ball-sports since a large amount of information must quickly be processed in a constantly changing environment (Vestberg et al., 2012, 2017, 2020). In line with those ideas, it has been suggested that measures of EF, and especially HEF, have an important role in soccer (Verburgh et al., 2014, 2016; Huijgen et al., 2015; Vestberg et al., 2017, 2020; Krenn et al., 2018; Sakamoto et al., 2018; Schumacher et al., 2018; Scharfen and Memmert, 2019) and other ball sports (Alves et al., 2013; Faubert, 2013; Wang et al., 2013, 2016; Lundgren et al., 2016; Alarcón et al., 2017; Liao et al., 2017; Ishihara et al., 2018; Wylie et al., 2018).

Another profession in which EF may be of fundamental importance is law enforcement. A police officer must deal with increasing demands for public safety under pressure from crime and terrorism (Gutshall et al., 2017). Police officers need to be able to handle a wide range of different situations, sometimes also under physical and even death threats. Since a police officer may encounter situations involving a large amount of information flow that is changing quickly (Gutshall et al., 2017), it may be hypothesized that well-functioning EF are a prerequisite for managing the profession, especially when there are time constraints and the stress level is high.

To be accepted to the police officers program, the applicant needs to meet specific requirements regarding physical, medical, and psychological status (Admission Requirements for Police Training, 2020). Medical obligations include absence of chronic somatic and psychiatric disorders. For the physical requirements, individuals must fulfill a specific level of physical work capacity and muscle strength. The psychological demands include a certain level of personal maturity, responsiveness, flexibility, commitment, and responsibility—as well as a well-developed communication skill. These requirements are assessed systematically before the individual is allowed to start in the police academy.

Significantly higher demands are required for joining an elite police force, such as the Swedish counterterror intervention unit [Nationella Insatsstyrkan (NI)] (Swedish Counterterror Intervention Unit, 2020). Their primary assignment is to deal with difficult situations related to terrorism and support other police units in cases like hostage-taking and robbery. The basic requirements for becoming a member of NI are considerably higher than for the regular police force (Halsius, 2007; Work at Swedish Counterterror Intervention Unit, 2020). Apart from high physical performance, such positions require individuals who can think independently, have a realistic sense of perception, and function well in groups—even under heavy stress (Halsius, 2007; Work at Swedish Counterterror Intervention Unit, 2020).

The admission process for NI is divided into different steps and takes several months to complete. The assessments are time-consuming and resource-demanding. They also require extensive physical and mental abilities (Werkelius, 1997). In the final step, the applicants will be closely observed during a 10-day extreme assessment test. The individual ability to use tactics, as well as being creative, work in teams, and perseverance, will be assessed under heavy stress (Werkelius, 1997; Halsius, 2007). Only a fraction of the applicants reaches the final level of testing, as most do not pass the earlier tests.

Situations of danger, that, for example, involve armed offenders, are associated with a high degree of stress, and may change and escalate quickly. It may be argued that individuals being recruited as operators for NI therefore should be able to cope optimally and find the most creative solutions in order to make the best decisions in such extreme circumstances. They should have a high capacity for cognitive flexibility to adjust dynamically in a quickly changing environment. They should also be resistant to stress and time constraints and inhibit some automatic behaviors that may be dangerous in specific contexts. The fast decisions made by an elite police officer may also result in unnecessary injuries and deaths of both police officers and offenders as well as in collateral casualties. Thus, both choices of most effective goal-directed behaviors and avoiding mistakes in such situations involve optimal EF apart from other cognitive abilities, emotional reactivity, and personality traits. Several of these requirements are similar to team sports, e.g., fast online problem-solving and high capacity to dynamically adapt in a quickly changing environment associated with an extensive information load. The military academy, a branch close to the police, also highlights *flexibility* paired with *creativity* as critical features for the military defense of the future (Finkel, 2011). The same reasoning may be applied for future successful elite police officers.

Previous research on police officers has studied how working memory relates to successful shooting behavior (Kleider et al., 2010) and indicates that EF is vitally important in extreme situations. Research on military special forces has focused on how stress influences working memory and learning (Taverniers et al., 2010; Morgan et al., 2011; Gutshall et al., 2017) and suggests that stress decreases the capacity of CEF. Studies on general population also suggest that lack of sleep (Yoo et al., 2007; Killgore, 2010; Lim and Dinges, 2010; Ma et al., 2015; Krause et al., 2017; Floros et al., 2020) and cognitive tiredness (Blain et al., 2016) have a negative impact on cognition, including EF. However, it is not clear how HEF capacity (including cognitive flexibility and creativity) changes when elite officers are under heavy physical and psychological pressure and sleep-deprived. Moreover, EF and HEF capacity of elite police officers has not been studied in relation to ordinary officers.

In the present study, we suggest that the ability to dynamically adapt in a rapidly changing environment involving a large amount threat is a core aspect for successful behavior both in the stress test that elite police applicants undergo and in situations where elite police officers may encounter a potential danger such as armed offenders or hostage taking. Arguably, such behavior should be associated with high EF capacity, especially HEF, as these cognitive abilities are fundamental for quickly solving complex problems. Performance in these contexts must also be malleable to mentally and physically stressful and exhausting situations, possibly affecting individuals with low EF capacity most. Based on these ideas, we more specifically hypothesized that: (1) selected applicants to the final field test have better HEF than regular police officer trainees and the normal population, (2) cognitive capacity of HEF would decline during a physical and psychologically stressful period, and (3) NI applicants with best baseline results would also have the best results in a stressful situation. We were especially interested in design fluency (DF) capacity as it has previously been shown to be a suitable test for successful behavior in a quickly changing environment where creative solutions are needed (Vestberg et al., 2012, 2017, 2020).

## Materials and Methods

### Ethics Statement

The study was approved by the local ethical committee (Regionala etikprövningsnämnden i Stockholm; Dnr 2015/528-31/4) and was performed in full compliance with the Declaration of Helsinki. All subjects were given verbal and written information on the study and gave their verbal and written informed consent to participate.

### Participants

Forty-five subjects were recruited to the *test-group* (NI applicants, *NIA*) and 30 subjects were recruited to the *control-group* (police officer trainee, *POT*).

Applicants to NI are required to have completed police and police aspirant training. Additionally, the applicant should have good eyesight, be well-trained, and have an aptitude for tactics and shooting. The *NIA-group* (*n* = 45; 44 men and one woman; Age range 27–41 years; *Mean age* = 31.7 years, SD = 3.33) consisted of applicants who passed psychological and physical screening tests, to be admitted to the counterterror intervention unit (NI). All applicants and their test results from the baseline testing were used. No subjects were excluded or failed in the baseline test situation. Several of the applicants failed the extreme field assessment test before the different re-tests of EF were performed and, thus, fewer subjects were re-tested (Re-test 1: *n* = 40, Re-test 2: *n* = 39, and Re-test 3: *n* = 36).

The *POT-group* (*n* = 30; 24 men and six women; Age range 22–39 years; *Mean age* = 27.7 years, SD = 4.70) consisted of participants who came from the basic training of police officers in the police academy of Stockholm. The school management asked two police classes (*N* ∼ 70), whether they would like to participate in the study. Out of these individuals, 30 police students chose to participate in the study. The *POT-group* was slightly but significantly younger in average than the test-group [*t*(73) = 4.365, *p* < 0.001].

### Materials

In the present study, we used tests from two test batteries: *The Delis–Kaplan EF System* (*D-KEFS*) *test battery* (Delis et al.,2001a,b; Shunk et al., 2006) and *The CogStateSports* (CS) *computerized concussion testing* (re-brand as Axon Sport) (Collie et al., 2003; Straume-Naesheim et al., 2005). All tests included in the present study have been standardized to a normal population of different age spans and sex.

### D-KEFS

*Delis–Kaplan EF system* measures different aspects of EF, and the subtests used in this study were DF, *Color-Word Interference Test*, and *Trail Making Test* mirroring our previous studies on EF in ball sports (Vestberg et al., 2012, 2017, 2020). D-KEFS is used in clinical assessments of patients, and there are well-described norms for the general population (Delis et al.,2001a,b; Shunk et al., 2006).

Design Fluency measures online multi-processing, including creativity, response inhibition, and cognitive flexibility (Homack et al., 2005; Swanson, 2005). *DF* is a non-verbal psychomotor test where dots are combined in a square with four lines using a pen. In *Condition 1*, the subject has to find as many different combinations as possible of binding together filled dots under time pressure (60 s). The subject is not allowed to use the same solution twice. The subject has to remember previous responses in a working memory and update it with new rules (i.e., not repeat previous combinations). He/she must use response inhibition in order not to repeat responses. The participant also needs to use scanning to find new possible solutions. In *Condition 2*, unfilled dots have been added into the square, and the subject should combine them with lines as in Condition 1. The filled dots are still present, but the participant should not use them in the task. In *Condition 3*, both filled and unfilled dots are present in the square. The task is to connect lines as above but also to switch between the filled and unfilled dots. The test is demanding due to its requirement to use creativity and cognitive flexibility (Delis et al., 2001a). A combination score (*DF Total Correct*) of the three subtests of *DF* was used as previously (Vestberg et al., 2012), to represent HEF (Vestberg et al., 2017, 2020) and capture both “simple creativity and fluency” and “advanced creativity” (with a higher demand on both inhibition and cognitive flexibility) mirroring the variability of problem solutions needed in action (Delis et al., 2001a). We also explored the results from the different subtests (*DF1, 2*, and *3*) as they differ in general demands on EF and cognitive flexibility. Especially, latent structure analysis has suggested that specifically DF3 involves a switching component suggesting a substantial cognitive flexibility aspect (Karr et al., 2018).

As previously (Vestberg et al., 2012, 2020), we used additional EF tests including *Trail Making Test* (*TMT*) (NIA and POT) and *Color Word Interference* (*CWI*) (results only for NIA) (Czerniak et al., 2015) as additional exploratory tests of general EFs, since they do not include the creativity and problem-solving components present in *DF* that was the main focus of this study.

### CogStateSports

*CogStateSports* (*CS*) is a non-verbal psychomotor test battery measuring basic attention, cognitive process speed, decision-making, speed and accuracy of short-term memory, and encoding of working memory (Collie et al., 2003; Straume-Naesheim et al., 2005). The subjects are shown different play cards on a computer screen and have to react as fast and correct as possible using different key responses. In the first test (“*Processing speed*”), the subject has to respond to any card that is displayed measuring simple response time. In the second test (“*Attention*”), the subject has to respond whether the card is red or black. This test measures simple attention. In a third test (“*Learning*”), the subject has to respond if he or she has seen the displayed card any time earlier in the test sequences. The test measures of more demanding working memory and learning. In the fourth test (“*Working memory*”), the subject has to decide if the previous card is the same as the card before, a measure of simple working memory (i.e., one-back memory-test).

### Procedure

The baseline testing (including all tests described above) of the NIA-group was performed in a standardized procedure in a quiet and secluded environment from May till June 2015. The POT-group was similarly assessed with the same tests from January till October 2016. A re-test of DF, TMT, and CWI was performed on the NIA-group in the extreme field assessment approximately 14 days later at different days for the different tests (Day 1: DF; Day 2: TMT; and Day 3: CWI). In the extreme field assessment, the applicants were pushed to their limits physically and mentally; meanwhile, the individuals were tested in their ability to handle the pressure, function in a group and to make adequate decisions. Importantly, all applicants went through the same stress tests at the same timepoints. The *NIA-group* and the *POT-group* had different test assessors.

### Statistical Analysis

Data were analyzed using IBM SPSS Statistics 25.0.0. Shapiro–Wilk test was used to test distributions for normality. Levene’s test was used to test the homogeneity of variances between the groups.

#### Hypothesis Testing Focusing on DF

An *ANCOVA* was used to compare the results of the two groups (The *NIA-group* vs. the *POT-group*) after adjusting for age and sex. We further used a *paired sample T-test* to assess if subjects in the *NIA-group* performed differently at the re-test (during field assessment) as compared to baseline testing. We used *Pearson’s correlation* to test for the relation between baseline scores and re-test scores. For any test (*DF Total correct*, *DF1, 2, or 3*) that showed a difference between baseline testing and the re-test, we also tested for the relation between baseline scores and percent drop in test results during the re-test, adjusting for the cognitive support functions as *Processing speed*, *Working memory* accuracy, and *Resting heart rate* (as a proxy for physical fitness; Sandvik et al., 1993; Jensen et al., 2013). *One-sample T-tests* were used to compare the main cognitive test results (*DF Total Correct* as well as its sub-components *DF1, DF2*, and *DF3*) of the *NIA-group* and the *POT-group* with the D-KEFS norm.

#### Additional Exploratory Analyses Focusing on Other EF-Tests

*Independent T-tests* were used to compare the *NIA-group* with the *POT-group* concerning the additional exploratory tests presented above. We also performed an exploratory-paired *sample T-test* to examine whether the other D-KEFS tests were different at baseline vs. the re-test sessions.

## Results

Our specific hypotheses pertained to the results from the *DF total Correct* and its subtests for NIA compared to the control group in the baseline assessment, as well as the results of NIA in baseline assessment compared to the field assessment. Levene’s test indicated equal variances assumed for the dependent variable across the groups. The model assumptions were met using Shapiro–Wilk test for normality of the residuals.

### Comparison Between NIA and POT

#### DF Total Correct

**FIGURE 1:**
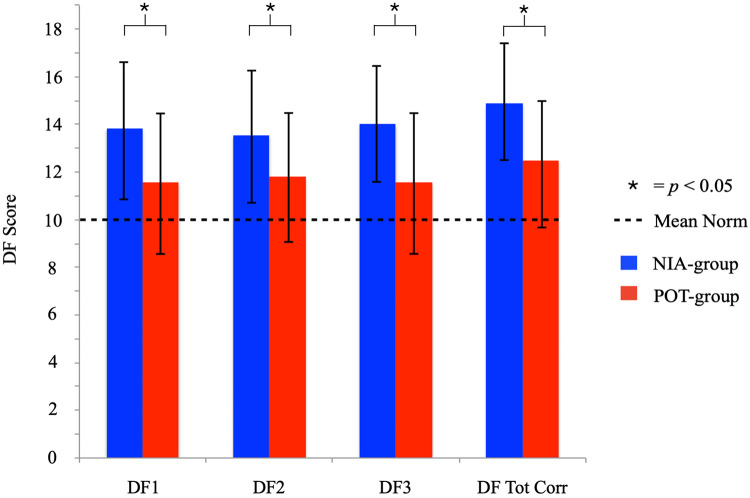
Mean and standard deviation (SD) of all the DF subtests (DF1, DF2, DF3) and the DF total score (DF Total C) in NIA-group and the POT-group.

We used a general linear model with the result of *DF Total Correct* as dependent variable and group, age, and sex as independent variables. We found that there was a large effect (Cohen’s *d* = 1.03) between groups, *F*(1,71) = 18.98, *p* < 0.001, η*_p_*^2^ = 0.21, suggesting that *NIA-group* performs better than *POT-group*. Age and sex did not have a significant effect on the score of *DF Total Correct* [Age, *F*(1,71) = 3.17, *p* = 0.079, η*_p_*^2^ = 0.043; Sex, *F*(1,71) = 0.37, *p* = 0.55, η*_p_*^2^ = 0.005]. The mean values of the *NIA-group* and the *POT-group* are presented in Figure 1. Since few female subjects were included in the two groups (one in the *NIA-group* and six in the *POT-group*), we also performed the analyses with only male subjects. As expected, the results were similar (see Supplementary Material).

#### DF Subtests

We found that there was a significant effect of group for DF1 [*F*(1,71) = 11.06, *p* < 0.001, η*_p_*^2^ = 0.14; Cohen’s *d* = 0.79], DF2 [*F*(1,71) = 6.97, *p* < 0.01, η*_p_*^2^ = 0.089; Cohen’s *d* = 0.63], and DF3 [*F*(1,71) = 22.66, *p* < 0.001, η*_p_*^2^ = 0.24; Cohen’s *d* = 1.12], suggesting that *NIA-group* performs better than *POT-group*. Age and sex did not have a significant effect on the scores, except for DF3 where there also was a significant effect of Age [*F*(1,71) = 4.76, *p* < 0.032, η*_p_*^2^ = 0.063].

### Baseline Assessment Compared to the “Field” Assessment in NIA

#### DF Total Correct

A paired sample *t*-test indicated that *DF Total Correct* scores were not significantly different between baseline assessment (*M* = 14.98, *SD* = 2.44) and the “field” assessment (*M* = 15.30, *SD* = 2.27), *t*(39) = −0.86, *p* = 0.39. There was a significant correlation between the results of the baseline assessment and the results obtained during the “field” assessment, *r*(40) = 0.49, *p* = 0.001.

#### DF Subtests

##### DF1

A paired sample *t*-test indicated that *DF1* scores were significantly different between baseline assessment (*M* = 13.95, *SD* = 2.84) and the re-test [*M* = 15.25, *SD* = 2.84; *t*(39) = −0.2.53, *p* = 0.016] suggesting an improvement of test results by approximately 9.3%. There was a significant correlation between the results of the baseline assessment and the re-test [*r*(40) = 0.35, *p* = 0.029].

##### DF2

A paired sample *t*-test indicated that *DF2* scores were not significantly different between baseline assessment (*M* = 13.68, *SD* = 2.67) and the re-test [*M* = 13.98, *SD* = 1.78; *t*(39) = −0.8, *p* = 0.43]. There was a significant correlation between the results of the baseline assessment and the re-test [*r*(40) = 0.5, *p* = 0.001].

##### DF3

A paired samples *t*-test indicated that the scores from *DF3* were significant different between baseline assessment (*M* = 14.2, *SD* = 2.38) compared with re-test [*M* = 12.83, *SD* = 2.23; *t*(39) = 3.79, *p* = 0.001] suggesting a worsening of test results by approximately 9.6%. There was also a significant correlation between the results of the baseline assessment and re-test [*r*(40) = 0.49, *p* = 0.001].

### Relation Between Baseline and Re-test of DF3

**FIGURE 2:**
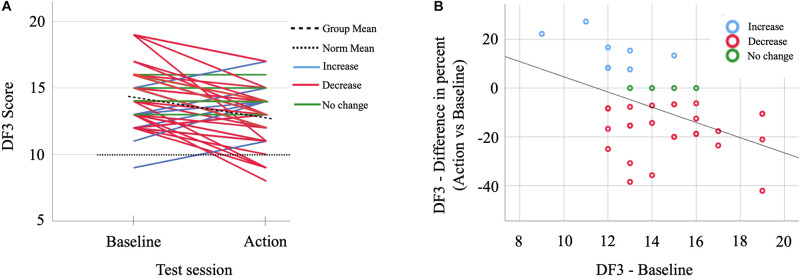
**(A)** The DF3 results from the baseline measurements and from the stress test for all NIA participants. **(B)** The difference in DF3 results between baseline and stress test in percent change as compared to baseline scores for all NIA participants.

In order to better understand the results above suggesting a worsening between DF3 results at baseline and at the re-test, we explored whether the subjects that had the best *DF3* result at baseline also had the smallest reduction in performance during the field assessment (re-test). We therefore first correlate the baseline *DF3* results for each individual with their baseline vs. re-test difference in percentage. We observed a significant negative correlation *r*(40) = −0.46, *p* = 0.003, i.e., higher DF3 results at baseline indicate a higher drop in the re-test result. This effect remained when we adjusted for *Process speed*, *Working memory*, and *Resting heart rate* as independent variables in an Ancova [*F*(1,35) = 9.16, *p* = 0.005, η*_p_*^2^ = 0.21; Cohen’s *d* = 1.03]. The cognitive capacity including *Process speed* and *Working memory* as well as *Resting heart rate* (as a proxy for physical fitness; Sandvik et al., 1993; Jensen et al., 2013) did not have any significant effect on the result. Thus, the subjects with the highest scores on *DF3* had the most dramatic fall of the results in the re-test performed during the extreme condition. However, subjects with higher results in baseline often still displayed high scores in the re-test assessment (Figures 2A,B).

### Exploratory Tests

Results of the comparison with the norm, as well as the results for the other performed tests, are of exploratory nature in order to generate hypotheses for further studies (see Supplementary Material). In general, those results suggest that both groups were better than the norm in the performed tests (except for TMT4 and CS Processing speed for the *POT*-group). In all tests where the two tested groups were compared, the *NIA-*group outperformed the *POT*-group significantly. Finally, while *NIA*-group became significantly worse in CWI3 during re-test, there was a non-significant trend that indicated that they became better in CWI4 and TMT4 during the re-test assessment. Moreover, there was a positive correlation between baseline test results and re-test results from the extreme field assessment for TMT4, CWI3, and CWI4 in the NIA-group.

## Discussion

The present study on elite police applicants forwards previous results on cognition of elite players in ball sports (Vestberg et al., 2012, 2017, 2020; Verburgh et al., 2014, 2016; Huijgen et al., 2015; Lundgren et al., 2016; Alarcón et al., 2017; Ishihara et al., 2018; Sakamoto et al., 2018) and suggests that *EF*s, and especially HEFs, also are decisive in other professions involving rapid flexibility and creative decision-making. In line with our initial hypothesis, the police officers that have passed all the tests until the last stage of the draft for the Swedish counterterror intervention unit, i.e., the *NIA-group*, were significantly better in the *DF test* than control group (*POT-group*). The results suggest that HEF may be of vital importance for becoming an elite police officer. Our additional exploratory analysis (Supplementary Material) also indicated that both police groups were somewhat better than the norm in test mirroring the CEF capacity but with a smaller effect size compared with DF and the HEF results. Although both groups were better than the norm in general attention tests from CogSport as well as TMT from D-KEFS, the *NIA-group* was significantly better than the *POT-group* in those tests (except for Learning). Altogether, the present results were similar to our previous study of adult elite and semi-elite soccer players (Vestberg et al., 2012) as well as when we studied two levels of play in elite players (Vestberg et al., 2020) in that the higher level players were better than the lower level players on DF, but both groups showed better results than the norm. The overall picture suggests that HEF is related to police officers able to past the steps in the selection to NI. One interpretation of this is that demanding EF and fast alternating and multifaceted creativity plays a vital role in the recruitment of the Swedish counterterror intervention unit (NI).

Comparing baseline *DF* results for the *NIA-group* with the results on the same test in a re-test “field” assessment when the subjects were under substantial physical and psychological stress suggests a decline in cognitive function. The technical manual of D-KEFS (Delis et al., 2001b) indicates that an expected increase of the results between the baseline and the re-test should be approximately 15% due to loss of the novelty factor. Here, the results for *DF Total Correct* only showed an average increase of 2%. When specifically analyzing the subtests, a corresponding increase was observed in a *DF1*, but the results for *DF3* showed an average 9.6% decrease of the result. This suggests that physical and psychological pressures most severely affect *cognitive flexibility*, which is the component that is most evident in *DF3* as compared to the other subtests of *DF* (Karr et al., 2018). Alternatively, the observed results may be due to the fact that *DF3* requires higher general focus than the other subtests.

Interestingly, the results show, contrary to our hypothesis, that the subjects with the highest baseline performance lost more of the capacity, in percent, than the subjects with low or moderate baseline performances. However, our result also suggests a positive correlation between baseline and the re-test assessment, and several of the individuals with the highest performance in the baseline test still had the some of the best results in the field test. Our interpretation of this result is that high baseline capacity of EF may also predict high capacity when a subject is under stress, although some high-performers have more difficulty reaching their maximum capacity when they are under heavy pressure. This is in line with the idea of an inverted U-cure of prefrontal top-down cognitive capacity (Cools and D’Esposito, 2011). Lower cognitive functions like *process speed* and *working memory*, as well as *resting heart rate* (as a proxy for physical fitness), did not have any effect on this result. The finding suggests that EF should be tested both during baseline conditions and during stressful conditions in order to fully describe an individuals’ cognitive capacity.

The relation between the present results and previous research on elite players of ball sport is striking (Vestberg et al., 2012, 2017, 2020; Verburgh et al., 2014, 2016; Huijgen et al., 2015; Lundgren et al., 2016; Alarcón et al., 2017; Ishihara et al., 2018; Sakamoto et al., 2018). Successful ballplayers must quickly adapt, change strategy, and inhibit responses—even after extreme tiredness and stress. Many of these abilities are referred to as “game intelligence” in sports (Stratton et al., 2004). We have argued that such abilities would essentially equal EF in a neuropsychological framework (Vestberg et al., 2012, 2017). The fast and creative problem-solving together with dynamic adaption due to quick changes on the soccer field suggests that HEF are especially important. Altogether, it seems that some of the behaviors required reaching an elite level in ball sports overlap with the requirements of elite police officers.

The importance of EF in police tasks has previously been suggested. For example, it has been shown that the capacity of the working memory correlates with successful shooting behavior among polices officers (Kleider et al., 2010). In a computerized test situation, officers with low working memory capacity shot more unarmed targets and fewer armed targets than officers with high capacity of the working memory. Controlling for working memory speed did not influence the result. This result indicates that working memory high capacity in form of accuracy is essential for successful behavior in armed police shooting interventions. Interestingly, the Swedish Police force seldom uses tests that objectively measure CEF or HEF, including creative and flexible problem-solving in action. Still, individuals with stronger HEF seem to do better in the required procedure or, alternatively, are more interested in applying for the position.

Working memory and other EF may be easily affected by stress and negative emotions (Taverniers et al., 2010), and a consequence of a declined working memory capacity seems to increase errors (Kleider et al., 2010; Taverniers et al., 2010; Gutshall et al., 2017). Likewise, it has been suggested that lack of sleep (Yoo et al., 2007; Killgore, 2010; Lim and Dinges, 2010; Ma et al., 2015; Krause et al., 2017; Floros et al., 2020) and mental tiredness (Blain et al., 2016) may also have a negative effect on EF. All of these potential negative effects on EF may be relevant for elite police force officers. Possibly, a higher baseline EF capacity may form a buffer that could prevent a critical functional deficit in an extreme situation (Floros et al., 2020).

In the present study, we did not measure IQ as we were specifically interested in the capacity to dynamical adapt in a quickly changing environment, a behavior not captured by general intelligence measurements. It has been suggested that HEF and fluid intelligence are synonymous (Diamond, 2013). However, while there is some correlation between EF-tests and tests of fluid intelligence (Conway et al., 2002; Egger et al., 2011; van Aken et al., 2016) especially when it comes to updating and working memory (Friedman et al., 2006; Friedman and Miyake, 2017; Krumm et al., 2018), the relationship is not complete (Conway et al., 2002; Egger et al., 2011; van Aken et al., 2016). The relation between EF and crystalized intelligence is substantially lower (Conway et al., 2002; Egger et al., 2011; van Aken et al., 2016). In line with this, we have previously shown that while EF-test correlated with measurement of successful behaviors, Raven’s matrices (Roca et al., 2010), a test that often is used as a proxy for fluid intelligence, did not (Vestberg et al., 2017). IQ and/or general mental ability (*g*) are like the concept of fluid intelligence and crystallized intelligence only to some aspects related to EF (Ardila et al., 2000; Friedman et al., 2006; Friedman et al., 2008; Wingo et al., 2013; Ardila, 2018). Nonetheless, although there is a separation between EF and IQ, IQ probably represents a unique component in successful behavior of elite police officers that need to be investigated.

Apart from EF and IQ, other cognitive functions such as emotional regulation could be important for elite police forces acting in quickly changing and potentially harmful environments. Although emotional regulation may be partially separated from EF, it represents similar top-down regulatory mechanisms, and the capacities of EF and emotional regulation are highly correlated (Petrovic and Castellanos, 2016). Therefore, measuring EF capacity may serve as a proxy for emotional regulation capacity of an individual. Moreover, social cognition abilities, spatial cognition, perceptual capacities, and grit may also be of importance for a successful function of elite police officers. Similarly, the predictive validity of personality traits should be investigated. Tedeholm et al. (2021) showed that individuals from Swedish counterterror intervention unit had lower results on the neuroticism scale and higher results on the conscientiousness and the extraversion scale than the norm in Swedish population (Tedeholm et al., 2021). However, in contrast to measurements of cognitive capacity, personality tests are subjective and their relation to behavior is still not fully evaluated.

A better understanding of the role of EF and how they shape successful behaviors in a constantly changing environment may strengthen future recruitment of elite police officers to become more effective in finding the right individuals suitable for the position. The role of the EF as a buffer to prevent critical functional deficits in cognitively stressful and changing environments needs to be studied further.

### Limitations

The subjects in the *NIA-group* were in average older than the subjects in the *POT-group*, possibly suggesting that life experience and more experience as a police officer could contribute to the results. However, the *ANCOVA* showed that age did not have any major effect on the results. Moreover, few female subjects were part of the tested groups and the groups also differed in number of female subjects (only one female subject was part of the tested *NIA-group* and six were part of the *POT-group*). This may have an effect on the results. However, we both controlled for sex in our main model when we compared groups and performed a supplementary analysis with male subjects only that did not change the main conclusions (presented in Supplementary Material). The comparison results between the *NIA-group* and the *POT-group* are not adjusted for the participants’ physical advantage. So we cannot exclude that it could affect the results. However, in the exploratory part of the study, we adjust for resting heart rate as a proxy for physical fitness. This adjustment did not have any significant impact on the EF results. We have not controlled for IQ, a measurement that, in general, is used in recruitment for police officers. Due to this, we cannot rule out that IQ may impact on the results. However, because of the weak relation between IQ and EF (Ardila et al., 2000; Conway et al., 2002; Friedman et al., 2006, 2008; Roca et al., 2010; Egger et al., 2011; Wingo et al., 2013; van Aken et al., 2016; Friedman and Miyake, 2017; Vestberg et al., 2017; Ardila, 2018; Krumm et al., 2018), this would be unlikely. The impact of other cognitive factors and personality on successful elite police behavior was not measured in the present study and should be assessed in the future. In the study, we used two assessors, one for each group. This could potentially confound the results comparing the *NIV*-group with the *POT*-group. Even if the assessors are trained to give the same instructions and use the tests in the same way, there may be differences between them. Notably, the *NIA*-group had better mean results than the *POT*-group in all performed tests, including the CogSport tests that are computer-based and need no verbal instructions, guidance, or assessment by a supervisor suggesting small assessor confound.

## Data Availability Statement

The datasets presented in this article are not readily available because some data contain classified information. Requests to access the datasets should be directed to PP, predrag.petrovic@ki.se.

## Ethics Statement

The studies involving human participants were reviewed and approved by Regionala etikprövningsnämnden i Stockholm; Dnr 2015/528-31/4. The patients/participants provided their written informed consent to participate in this study.

## Author Contributions

TV and PT tested subjects. TV, PT, and PP analyzed the data. All authors wrote the manuscript and were involved in designing and planning the study. All authors contributed to the article and approved the submitted version.

## Conflict of Interest

TV and PP have been involved as consultants in cognitive testing outside research. PP has stocks in company that tests cognition. The remaining authors declare that the research was conducted in the absence of any commercial or financial relationships that could be construed as a potential conflict of interest.
