# Supplementary material for: Executive Functions of Swedish Counterterror Intervention Unit Applicants and Police Officer Trainees Evaluated With Design Fluency Test
Source: Front Psychol. 2021 May 11;12:580463. doi: 10.3389/fpsyg.2021.580463 (PMC8185326; doi:10.3389/fpsyg.2021.580463)
Supplement: Supplementary file 1 [file Data_Sheet_1.DOCX]

**Executive functions of Swedish Counterterror intervention unit applicants and police officer trainees evaluated with design fluency test**

**- Supplemental materials**

Torbjörn Vestberg^a*^ , Peter Tedeholm^b *^, Martin Ingvar^a^, Agneta Larsson^b^ **

and Predrag Petrovic^a^**

^a^ = Department of Clinical Neuroscience, Karolinska Institutet, Stockholm, Sweden

^b^ = Department of Physiology and Pharmacology, Karolinska Institutet, Stockholm, Sweden

* = Contributed equally as first authors to the present study

** = Contributed equally as last authors to the present study

**Corresponding author:**

Predrag Petrovic

Department of Clinical Neuroscience

Karolinska Institutet

Nobels väg 9, 17165 Stockholm, Sweden

e-mail: [predrag.petrovic@ki.se](mailto:predrag.petrovic@ki.se)

**Design Fluency - Comparison with normal population**

**DF total correct**

Using the result of *DF Total Correct* in *one-sample t-test*, both the police officers in the *NIA-group* and the *POT-group* performed significantly above the normal population. The *NIA-group:* (*M*=14.87, *SD=2.54),* *t*(44) = 12.9, *p* < 0.001, (Cohen’s *d* = 1.92). The *POT-group:* (*M*=12.47, SD=2.6), *t*(29) = 5.15, *p* < 0.001, (Cohen’s *d* = 0.94).

**DF subtests**

We also tested the subtests (*DF1, 2* and *3*) as they differ in general demands on EF and cognitive flexibility.

**DF1:** Using *DF1,* in a *one-sample t-test*, both the police officers in the *NIA-group* and the *POT-group* performed significantly above the normal population *(NIA-group*: *M*=13.84, *SD=2.91,* *t*(44) = 8.85, *p* < 0.001, *d* = 1.32; *POT-group*: *M*=11.57, SD=2.96, *t*(29) = 2.9, *p* = 0.007, *d* = 0.53).

**DF2:** Using *DF2*, in a *one-sample t-test*, both the police officers in the *NIA-group* and the *POT-group* performed significantly above the normal population *(NIA-group*: *M*=13.56, *SD=2.7,* *t*(44) = 8.83, *p* < 0.001, *d* = 1.32; *POT-group*: *M*=11.8, SD=2.6, *t*(29) = 3.78, *p* < 0.001, *d* = 0.69).

**DF3:** Using *DF3*, in a *one-sample t-test*, both the police officers in the *NIA-group* and the *POT-group* performed significantly above the normal population *(NIA-group*: *M*=14.04, *SD=2.43,* *t*(44) = 11.16, *p* < 0.001, *d* = 1.25; *POT-group*: *M*=11.57, SD=2.9, *t*(29) = 2.9, *p* = 0.006, *d* = 0.54).

**Design Fluency - Comparison between groups**

See main text for the main results on DF total correct

**DF total correct - only males**

We used a general linear model with the result of *DF Total Correct* as dependent variable and group and age as independent variables. We found that there was a large effect (Cohen’s *d* = 1.025) between groups, *F*(1, 65) = 17.07, *p* < 0.001, *η_p_ ^2^* = 0.208, suggesting that *NIA-group* perform better than *POT-group*. Age did not have a significant effect on the score of *DF Total Correct (*Age, *F*(1, 65) = 2.6, *p* = 0.112, *η_p_ ^2^* = .038.

**DF subtests**

We found that there was a significant effect of group for **DF1** (*F*(1, 65) = 9.19, *p* < 0.004, *η_p_ ^2^* = 0.124; Cohen’s *d* = 0.75), **DF2** (*F*(1, 65) = 7.74, *p* < 0.007, *η_p_ ^2^* = 0.106; Cohen’s *d* = 0.69) and **DF3** (*F*(1, 65) = 21.6, *p* < 0.001, *η_p_ ^2^* = 0.249 (Cohen’s *d* = 1.15) suggesting that *NIA-group* perform better than *POT-group*. Age did not have a significant effect on the scores, except for DF3 where there also was a significant effect of Age (*F*(1,65) = 5.9*, p < 0.018, η_p_ ^2^* = 0.083.

**Additional exploratory tests**

**Comparison with normal population**

**CWI1-2:** Using the combination score of condition *CWI1* and CWI*2*, in a one-sample t-test, the police officers in the *NIA-group* performed significantly above the normal population *(NIA-group*: *M*=11.09, *SD*=1.52*,* *t*(43) = 475, *p* < 0.001, *d* = 0.72.

**CWI3:** Using *CWI3* in a one-sample t-test, the police officers in the *NIA-group* performed significantly above the normal population *(NIA-group*: *M*=12.59, *SD*=1.53*,* *t*(43) = 11.23, *p* < 0.001, *d* = 1.69.

**CWI4:** Using *CWI4* in a one-sample t-test, the police officers in the *NIA-group* performed significantly above the normal population *(NIA-group*: *M*=12.33, *SD*=1.19*,* *t*(43) = 12.33, *p* < 0.001, *d* = 1.86.

**TMT2-3:** Using the combination score of condition *TMT 2* and *TMT 3*, in a one-sample t-test, both the police officers in the *NIA-group* and the *POT-group* performed significantly above the normal population *(NIA-group*: *M*=13.47, *SD=1.87,* *t*(44) = 12.46, *p* < 0.001, *d* = 1.86; *POT-group*: *M*=12.1, SD=1.83, *t*(29) = 6.3, *p* < 0.001, *d* = 1.15).

**TMT4:** Using *TMT4* in a one-sample t-test, the police officers in the *NIA-group* performed significantly above the normal population, while the *POT-group* did not (*NIA-group: M*=11.6, *SD=1.72,* *t*(44) = 6.22, *p* < 0.001, *d* = 0.93; *POT-group: M*=10.5, SD=2.68, *t*(29) = 0.96, *p* = 0.35, *d* = 0.19)

**CS Processing speed:** Using *CS process* in one-sample t-test, the police officers in the *NIA-group*, but not the *POT-group*, performed significantly above the normal population (*NIA-group*: *M*=106.33, *SD=2.97,* *t*(44) = 14.31, *p* < 0.001, *d* = 2.12; *POT-group*: *M*=101.3, SD=11.3, *t*(29) = 0.65, *p* = .52, *d* = 0.12).

**CS Attention:** Using the *CS Attention*, in one-sample t-test, both the police officers in the *NIA-group* and the *POT-group* performed significantly above the normal population (*NIA-group*: *M*=108.53, *SD=3.04,* *t*(44) = 18.83, *p* < 0.001, *d* =2.8; *POT-group*: *M*=106.09, SD=5.34, *t*(29) = 6.25, *p* < 0.001, *d* = 1.14).

**CS Working memory (WM):** Using *WM*, in one-sample t-test, the police officers in the *NIA-group* performed significantly above the normal population while there was a trend effect for the *POT-group* (*NIA-group:* *M*=105.17, *SD=4.35,* *t*(44) = 7.97, *p* < 0.001, *d* = 1.19; *POT-group:* *M*=102.4, SD=6.58, *t*(29) = 2.0, *p* = 0.055, *d* = 0.36)**.**

**CS Learning:** Using the result of *Learning* in one-sample t-test, both the police officers in the *NIA-group* and the *POT-group* performed significantly above the normal population. *NIA-group:* (*M*=105.4, *SD=5.9),* *t*(44) = 6.14, *p* < 0.001, *d* = 0.92; *POT-group:* (*M*=104.08, SD=6.04), *t*(29) = 3.7, *p* < 0.001, *d* = 0.68.

**Comparison between the NIA-group and the POT group**

**TMT2-3:** An independent-sample t-test indicated that the combination scores of condition TMT 2 and 3 were significant higher for the *NIA-group* (*M*=13.47, *SD=1.87)* than for the *POT-group* (*M*=12.1, SD=1.83), *t*(73) = 3.1, *p* = 0.002, *d* = 0.74.

**TMT4:** An independent-sample t-test indicated that the TMT 4 scores were significant higher for the *NIA-group* (*M*=11.60, *SD=1.72)* than for the *PT-group* (*M*=10.5, SD=2.68), *t*(45) = 2.06, *p* = 0.046, *d* =0.49. Levene’s test indicated unequal variances (F = 5.43, *p* = 0.023), so degrees of freedom were adjusted from 73 to 45.

**CS Processing speed:** An independent-sample t-test indicated that the *CS processing* *speed* scores were significant higher for the *NIA-group* (*M*=106.33, *SD=2.97)* than for the *POT-group* (*M*=101.3, SD=11.3), *t*(31,69) = 2.36, *p* = 0.025, *d* = 0.6. Levene’s test indicated unequal variances (F = 13,12, *p* = 0.001), so degrees of freedom were adjusted accordingly.

**CS Attention:** An independent-sample t-test indicated that the *CS Attention* scores were significant higher for the *NIA-group* (*M*=108.53, *SD=3.04)* then for the *POT-group* (*M*=106.1, SD=5.3), *t*(73) = 2.52, *p* = 0.014, *d* = 0.56.

**CS Working Memory (WM):** An independent-sample t-test indicated that the *WM* scores were significantly higher for the *NIA-group* (*M*=105.17, *SD=4.35)* than for the *POT-group* (*M*=102.4, SD=6.58), *t*(45.8) = 2.02, *p* = 0.049*, d =0.5).* Levene’s test indicated unequal variances (F = 5,82, *p* = 0.018), so degrees of freedom were adjusted accordingly.

**CS Learning**: An independent-sample t-test indicated that the *CS Learning* scores were not significant higher for the *NIA-group* (*M*=105.4, *SD=5.9)* then for the *POT-group* (*M*=104.1, SD=6.01), *t*(73) = 0.93, *p = 0.35*, *d* = 0.22.

**Supplementary Table 1. Relation between Baseline and Re-test of TMT4, CWI3 and 4**

| *Paired Samples Statistics* | | | | | |
| --- | --- | --- | --- | --- | --- |
|  | | Mean | N | Std. Deviation | Std. Error Mean |
| Pair 1 | TMT4Baseline | 11.82 | 39 | 1.502 | 0.241 |
|  | TMT4Action | 12.26 | 39 | 1.428 | 0.229 |
| Pair 2 | CWI3Basline | 12.75 | 36 | 1.402 | 0.234 |
|  | CWI3Action | 12.31 | 36 | 1.653 | 0.276 |
| Pair 3 | CWI4Baseline | 12.19 | 36 | 1.283 | 0.214 |
|  | CWI4Action | 12.61 | 36 | 1.315 | 0.219 |

| *Paired Samples Correlations* | | | | |
| --- | --- | --- | --- | --- |
|  | | N | Correlation | Sig. |
| Pair 1 | TMT4Baseline & TMT4Action | 39 | 0.402 | 0.011 |
| Pair 2 | CWI3Basline & CWI3Action | 36 | 0.786 | 0.001 |
| Pair 3 | CWI4Baseline & CWI4Action | 36 | 0.452 | 0.006 |

| *Paired Samples Test* | | | | | | | | | | | | | |  |  |
| --- | --- | --- | --- | --- | --- | --- | --- | --- | --- | --- | --- | --- | --- | --- | --- |
|  | | | | Paired Differences | | | | | | | | | | | |
|  | | Mean | | | Std. Deviation | | t | |  | | | |  |  |  |
|  |  |  |  |  |  |  |  |  | df | | Sig. (2-tailed) | | | |  |
| Pair 1 | TMT4Baseline - TMT4Action | | -0.436 | | | 1,603 | | -1.699 | | 38 | | 0.098 | |  |  |
| Pair 2 | CWI3Basline - CWI3Action | | 0.444 | | | 1.027 | | 2.598 | | 35 | | 0.014 | |  |  |
| Pair 3 | CWI4Baseline - CWI4Action | | -0.417 | | | 1.360 | | -1.838 | | 35 | | 0.075 | |  |  |
